# Proteomic Analysis of Dairy Cows with Persistent Subclinical Hypocalcemia

**DOI:** 10.3390/ani16152378

**Published:** 2026-08-03

**Authors:** Yunlong Bai, Junbo Hu, Jingyi Liu, Xudong Sun, Chuang Xu, Jiajing Liu, Xiaochen Jia, Yu Yang, Lianying Wang, Guang Shao, Qitao Zhu, Caixia Ru, Mengjiao Wang, Cheng Xia, Yuxi Song

**Affiliations:** 1 College of Animal Science and Veterinary Medicine, Heilongjiang Bayi Agriculture University, Daqing 163319, China; 2 College of Veterinary Medicine, China Agricultural University, Beijing 100193, China; 3 Heilongjiang Mudanjiang Agricultural Reclamation Jiangjun Dairy Cow Breeding Professional Cooperative, Jixi 158100, China; 4 Jiamusi Animal Disease Prevention and Control Center, Jiamusi 154000, China; 5 Daqing Agricultural and Rural Social Undertakings Service Center, Daqing 163319, China; 6 Branch of Animal Husbandry and Veterinary Sciences, Heilongjiang Academy of Agricultural Sciences, Qiqihar 161000, China; 7 Inner Mongolia Shengmu High-Tech Animal Husbandry Co., Ltd., Hohhot 010111, China; 8 Xi’an Caotan Animal Husbandry Co., Ltd., Xi’an 710018, China; 9 Hospital of Heilongjiang Bayi Agricultural University, Daqing 163319, China

**Keywords:** dairy cows, persistent subclinical hypocalcemia, proteomics, 4D-DIA quantitative proteomics

## Abstract

To delineate the serum proteomic profile of persistent subclinical hypocalcemia (pSCH) in periparturient dairy cows, elucidate its molecular pathogenesis, and provide a theoretical basis for early warning and precision prevention and control, we selected 12 Holstein dairy cows of similar age (2.92 ± 0.12 years), parity (1.56 ± 0.21), body condition score (BCS) (2.84 ± 0.04), milk yield (26.81 ± 0.22 kg/d), and day in milk (DIM) (6.60 ± 0.24 d) and no significant between-group differences as experimental animals. Based on serum calcium concentrations on postpartum days 1 to 4 and clinical presentation, the cows were divided into a healthy control group (serum calcium > 1.77 mmol/L on day 1 and >2.20 mmol/L on day 4 postpartum, *n* = 6) and a persistent subclinical hypocalcemia group (serum calcium ≤ 1.77 mmol/L on day 1 and ≤2.20 mmol/L on day 4 postpartum, n = 6). Serum samples were collected on postpartum days 1, 2, and 4 and analyzed using 4D-DIA quantitative proteomics. A total of 178 significantly differentially expressed proteins were identified (fold change > 1.2, *p* < 0.05), including 59 up-regulated and 119 down-regulated proteins. These differentially expressed proteins were mainly enriched in pathways involving endocrine and other factor-regulated calcium reabsorption, regulation of actin cytoskeleton, the tricarboxylic acid cycle (TCA cycle), and lipoic acid metabolism. The results indicate that the pathological state of pSCH is closely associated with abnormalities in calcium reabsorption regulation, actin cytoskeleton maintenance, and mitochondrial energy metabolism-related signaling pathways. The key proteins and pathways identified in this study provide a theoretical foundation for future in-depth research on the pathogenesis, prevention, and treatment of pSCH.

## Simple Summary

This study focuses on persistent subclinical hypocalcemia in postpartum dairy cows, a condition that presents no overt clinical signs yet elevates the risk of disease and culling, and whose pathogenesis remains unclear. By comparing serum protein profiles between healthy and affected cows, we identified 178 differentially expressed proteins and found that the disorder is closely associated with dysfunctions in calcium absorption, maintenance of cytoskeletal integrity, and energy metabolism. This work elucidates the protein-level pathogenic characteristics of the disease, provides a scientific basis for early prevention and control on dairy farms, and can help reduce disease risk.

## 1. Introduction

Dairy cows undergo profound physiological changes around parturition [1]. The onset of lactation drives an increased demand for calcium, causing blood calcium concentrations to drop abruptly within 2–3 days before and after calving [2], which exceeds the capacity of homeostatic regulation and results in hypocalcemia [3]. In addition, accelerated fetal skeletal calcification during late gestation markedly elevates maternal calcium output, continuously depleting body calcium reserves. During parturition, uterine smooth muscle contractions and skeletal muscle straining are highly dependent on calcium ions, consuming a large amount of circulating calcium within a short period. This calcium drain, combined with the calcium loss into colostrum synthesis, creates an additive effect that precipitates hypocalcemia in dairy cows. 

Approximately 2.2% of dairy cows develop clinical signs [4], whereas around 50% of multiparous cows experience subclinical hypocalcemia [5]. Although clinical cases are more severe, subclinical cases are difficult to detect early because they lack typical clinical manifestations, and they increase the incidence of other periparturient diseases [6]. Subclinical hypocalcemia in dairy cows is a common disorder linked to negative calcium balance during the first 3 d postpartum; affected cows typically have blood calcium concentrations between 1.38 and 2.00 mmol/L and show no overt clinical signs [7]. It has been reported that the daily calcium requirement of dairy cows at the onset of lactation increases by approximately 65% compared with the dry period; however, due to the combined effects of parturition stress and reduced feed intake, intestinal calcium absorption efficiency is markedly decreased at this stage, driving the animal into a negative calcium balance that precipitates both clinical and subclinical hypocalcemia [8,9]. In recent years, new research findings on the classification of hypocalcemia have been widely recognized and accepted in the field. McArt et al. (2020) proposed criteria for subclinical hypocalcemia that classify affected cows into three subtypes based on parity (primiparous vs. multiparous) and serum calcium concentrations on postpartum days 1 to 4 [10]: (1) Healthy cows (HCs): primiparous cows with serum calcium > 2.15 mmol/L on postpartum days 1 and 2; multiparous cows with serum calcium > 1.77 mmol/L on day 1 and >2.20 mmol/L on day 4. (2) Transient subclinical hypocalcemia (tSCH): primiparous cows with serum calcium ≤ 2.15 mmol/L on day 1 and >2.15 mmol/L on day 2; multiparous cows with serum calcium ≤ 1.77 mmol/L on day 1 and >2.20 mmol/L on day 4. (3) Delayed subclinical hypocalcemia (dSCH): primiparous cows with serum calcium > 2.15 mmol/L on day 1 and ≤2.15 mmol/L on day 2; multiparous cows with serum calcium > 1.77 mmol/L on day 1 and ≤2.20 mmol/L on day 4. (4) Persistent subclinical hypocalcemia (pSCH): primiparous cows with serum calcium ≤ 2.15 mmol/L on both days 1 and 2; multiparous cows with serum calcium ≤ 1.77 mmol/L on day 1 and ≤2.20 mmol/L on day 4. In terms of incidence, pSCH accounts for a considerable proportion of fresh cows and warrants close attention [9]. A study by McArt et al. involving 407 Holstein cows reported that the incidence of pSCH was approximately 13% in multiparous cows and as high as approximately 23% in primiparous cows [10]. Seely et al., using different calcium cutoffs (≤1.89 mmol/L on day 1 and ≤2.25 mmol/L on day 4), reported that pSCH accounted for 27% of 89 multiparous Holstein cows [9]. Ghasemi et al. further demonstrated that the proportion of pSCH is significantly higher in multiparous cows than in primiparous cows, indicating that in dairy practice, pSCH is not a rare occurrence and is particularly prevalent among multiparous cows, which bear the heaviest metabolic burden during peak lactation [11]. The detrimental impact of pSCH is substantially greater than that of the other two subtypes, posing a serious threat to cow health and performance. McArt and Neves showed that, compared with normocalcemic cows, cows with pSCH had a significantly higher risk of adverse events (ketosis, metritis, displaced abomasum, or removal from the herd within 60 days), with hazard ratios of 4.1 for primiparous cows and 1.8 for multiparous cows [10].

Research on the pathogenesis of pSCH remains largely lacking. In addition to intestinal absorption and skeletal mobilization, the kidney plays an indispensable role in the fine regulation of systemic calcium homeostasis through selective reabsorption of filtered calcium in the distal convoluted tubule and collecting duct, in which the transcellular calcium transport mediated by the apical calcium channel TRPV5 serves as the rate-limiting step and is directly regulated by various endocrine factors and endocytic recycling mechanisms [12]. Serum proteomics enables a systemic view of pSCH pathophysiology by capturing organ-derived functional proteins involved in calcium homeostasis, energy metabolism, and cytoskeletal integrity. The rationale for this approach lies in its capacity to uncover global molecular dysregulations beyond known biochemical markers. Biologically, these protein signatures reflect the integrated dysfunction of renal, mammary, and muscular systems. Clinically, given the accessibility of serum, identified differential proteins hold promise as early biomarkers to distinguish pSCH from transient hypocalcemia and to predict risks of secondary diseases and culling, thereby supporting precision herd health management. Therefore, the objective of this study was to characterize its proteomic profile, enrich the etiological understanding, and provide a basis for disease prevention and control. 

## 2. Materials and Methods

### 2.1. Measurement of Serum Calcium Concentration

All cows had ad libitum access to a total mixed ration (TMR). The basal TMR consisted of the following ingredients on an air-dry basis: soybean meal 8.0%, alfalfa hay 8.0%, beet pulp 2.7%, corn gluten meal 1.3%, oat hay 2.1%, high-moisture corn 13.3%, whole-plant corn silage 53.1%, premix 1.4%, sodium bicarbonate 0.8%, calcium bicarbonate 0.3%, fat powder 0.8%, cottonseed 2.6%, coated urea 0.3%, soybean hulls 2.6%, and molasses 2.7%. Each kilogram of premix contained: vitamin A 350,000 IU, vitamin D_3_ 85,000 IU, vitamin E 2000 IU, Cu 800 mg, Zn 2400 mg, Mn 1200 mg, Co 10 mg, Se 24 mg, I 20 mg, Ca 148 mg, and P 12 mg. Water was provided ad libitum, and cows were milked three times daily at 04:00, 12:00, and 18:00. From all experimental cows, 10 mL of blood was collected from the coccygeal vein before morning feeding on postpartum days 1, 2, and 4. Blood samples were drawn into disposable vacuum blood collection tubes, centrifuged at 4000 rpm for 10 min, and the supernatant was aliquoted into 1.5 mL microcentrifuge tubes. After a second centrifugation at 12,000 rpm for 5 min, the serum was aliquoted, snap-frozen in liquid nitrogen, and stored at −80℃ for subsequent proteomic analysis. This study involving experimental animals was approved by the Animal Ethics Committee of Heilongjiang Bayi Agricultural University (approval number DWKJXY2024017, approved on 10 May 2024). It complies with the U. S. National Institutes of Health Guide for the Care and Use of Laboratory Animals. The experiment was conducted on a large-scale intensive dairy farm in the eastern region of Heilongjiang Province, China. Twelve Holstein dairy cows with similar age (2.96 ± 0.10 years), parity (1.60 ± 0.09), body condition score (2.84 ± 0.02), milk yield (26.83 ± 0.25 kg/d), and days postpartum (6.62 ± 0.27 d) and no significant differences among groups were selected as experimental animals after clinical examination excluded other diseases. 

### 2.2. Grouping of Experimental Animals

Initially, a total of 30 Holstein dairy cows (including both primiparous and multiparous animals). Based on serum calcium concentrations within 1–4 days postpartum and clinical presentation, the cows were divided into two groups (n = 6 per group). For primiparous cows (parity = 1), persistent subclinical hypocalcemia (pSCH) was defined as serum calcium ≤ 2.15 mmol/L on both day 1 and day 2 postpartum, while healthy controls (HC) were those with serum calcium > 2.15 mmol/L on both days. Multiparous cows with serum calcium > 1.77 mmol/L on postpartum day 1 and >2.20 mmol/L on day 4 were designated as healthy cows, and those with serum calcium ≤ 1.77 mmol/L on day 1 and ≤2.20 mmol/L on day 4 were designated as pSCH cows [9]. Twelve cows free of any disease other than hypocalcemia were selected; Group A served as the healthy control (HC) group and Group B as the pSCH group. The identification numbers in Group A were N1, N2, N3, N4, N5, and N6, and those in Group B were P1, P2, P3, P4, P5, and P6. Blood samples were collected daily at scheduled times from each cow. Immediately after sampling, the cows were returned to the original herd and uniformly maintained under the farm’s routine feeding and management conditions. Serum collected on postpartum day 2 was used for 4D-DIA proteomic analysis. 

### 2.3. Protein Extraction and Enzymatic Hydrolysis

To precisely inhibit protease activity, the sample temperature was strictly maintained at 0–4 °C using an ice bath, a condition under which the activity of endogenous proteases is only 1–2% of that at 37 °C. Samples were thawed vertically on ice, vortexed, and 50 μL of the clarified supernatant was transferred to an automated 96-well plate. Using the Qinglian Low-Abundance Protein Enrichment Kit (Qinglian Bio-Technology Co., Ltd. Beijing, China), the sample was mixed with the dilution buffer (DMB-DB-2), added to pre-incubated DMB beads, and incubated at 37 °C with shaking at 1000 rpm for 30 min. The beads were magnetically separated, the supernatant was discarded, and the beads were washed three times to obtain magnetic beads carrying low-abundance proteins. Subsequently, 50 μL of digestion buffer (DMB-ESB-3) and 1 μL of 0.5 μg/μL protease (DMB-E-10) were added to the beads and incubated at the same temperature and shaking speed for more than 4 h. After magnetic separation of the supernatant, 25 μL of stop solution (DMB-SB-4) was added to terminate the digestion. 

For SDB desalting column cleanup, the sample was mixed with 50 μL of loading solution (DMB-LB-5) and 25 μL of stop solution, vortexed, and loaded completely onto the column. The column was washed sequentially with 100 μL of washing solution 1 (DMB-WB1-6) and 100 μL of washing solution 2 (DMB-WB2-7) to remove impurities, and finally eluted with 100 μL of elution buffer (DMB-EB-8). The eluate was collected and lyophilized. 

### 2.4. Peptide Fractionation

The pooled labeled sample was dissolved in 100 μL of mobile phase A and centrifuged at 14,000× *g* for 20 min. The supernatant was fractionated by high-performance liquid chromatography (HPLC). The chromatographic separation was performed at a flow rate of 0.7 mL/min using a linear gradient of mobile phase B, with the following program: 0–5 min, 5–8% B; 5–40 min, 8–18% B; 40–62 min, 18–32% B; 62–64 min, B was rapidly increased to 95% and held at 95% for column washing until 68 min; finally, 68–72 min, B was returned to 5% for column equilibration prior to the next injection. 

### 2.5. LC-MS/MS Mass Spectrometry Analysis

The lyophilized peptide powder was dissolved in 10 μL of mobile phase A (100% water with 0.1% formic acid) and centrifuged at 14,000× *g* for 20 min at 4 °C. A supernatant volume equivalent to 200 ng of peptides was injected for liquid chromatography–mass spectrometry (LC-MS) analysis. Chromatographic separation was performed using the Evosep One 40 SPD method with gradient elution; mobile phase B consisted of 80% acetonitrile containing 0.1% formic acid. Mass spectrometry analysis was conducted on a timsTOF Pro mass spectrometer equipped with a protein analysis column (catalog no. QL-HPLC-100×15, length 100 μm, inner diameter 1.5 μm, stationary phase 250 mm, Qinglian Bio-Technology Co., Ltd. Beijing, China) operated in data-independent acquisition (DIA) mode. The instrument was operated in positive ion mode, and both MS and MS/MS spectra were acquired over the mass range of 100–1700 *m*/*z*. MS2 data were collected using a DIA strategy with four TIMS scan windows, each with an accumulation time of 100 ms. In PASEF mode, the collision energy was linearly ramped as a function of ion mobility (1/K0). Raw mass spectrometry data were searched against the *Bos taurus* UniProt database using Spectronaut software with the following parameters: enzyme, trypsin; fixed modification, carbamidomethyl (C); variable modifications, methionine oxidation (+15.995 Da) and protein N-terminal acetylation; maximum missed cleavages, 2. 

### 2.6. Statistical Analyses

Blood biochemical parameters and cow performance data were analyzed using IBM SPSS 26.0. After confirming normal distribution (Kolmogorov–Smirnov test) and homogeneity of variances, differences in serum calcium concentrations between the two groups were assessed by one-way analysis of variance (one-way ANOVA). For proteomic data, spectra were searched against the *Bos taurus* UniProt database using Spectronaut for protein identification and quantification. The quantitative data were subjected to median normalization based on common proteins. Proteins with valid values in all samples were used to generate PCA plots comparing hypocalcemic cows with healthy controls via the ggbiplot package. After re-normalization, proteins with missing values in >50% of samples were filtered out, and remaining missing values were imputed using the K-nearest neighbor (KNN) method. Differentially expressed proteins were identified by pairwise *t*-test between groups, with a fold change > 1.2 and *p* < 0.05 set as the significance threshold. Multivariate analysis and orthogonal partial least squares discriminant analysis (OPLS-DA) were performed using SIMCA 14.1 (Umetrics AB, Umeå, Sweden). Potential overfitting of the OPLS-DA model was assessed by 200 permutation tests, with quality parameters including goodness of fit (R^2^Y) and predictive ability (Q^2^). Functional annotation of protein families and pathways was conducted using COG, KEGG, and Reactome databases. Gene Ontology (GO) annotation, covering molecular function, biological process, and cellular component, was derived from the QuickGO database. Pathway enrichment analysis of differentially expressed proteins was performed using the KEGG database with a hypergeometric distribution algorithm. Protein–protein interactions were predicted using the STRING database (http://string.embl.de/). The full names and definitions of all abbreviations used in this manuscript are listed in Supplementary Materials:

## 3. Results

As shown in Table 1, the background information of the two groups was as follows: age 2.92 ± 0.12 years, parity 1.56 ± 0.21, BCS 2.84 ± 0.04, milk yield 26.81 ± 0.22 kg/d, and DIM 6.60 ± 0.24 d. At enrollment (postpartum day 1), serum calcium concentrations already met the predefined grouping criteria: all healthy cows had concentrations > 1.77 mmol/L, whereas all pSCH cows had concentrations ≤ 1.77 mmol/L. On postpartum day 2, the between-group difference in serum calcium was most pronounced, and the disease phenotype was the most stable and typical. By postpartum day 4, serum calcium in the healthy group rebounded to above 2.20 mmol/L, while in the pSCH group it remained below 2.20 mmol/L, meeting the diagnostic criteria for persistent subclinical hypocalcemia. To maximally capture the differences in serum protein expression under the pathological state of pSCH and minimize interference from subsequent secondary metabolic disorders, we selected serum samples collected on postpartum day 2 for the subsequent 4D-DIA quantitative proteomic analysis. 

### 3.1. Multivariate Statistical Analysis

**Figure 1:**
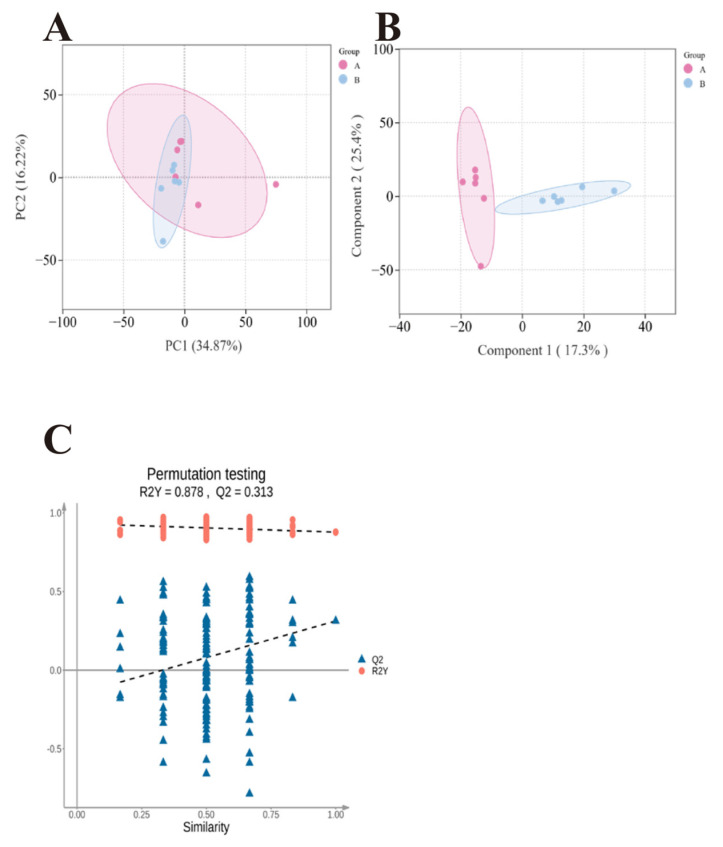
Panel (**A**) shows the PCA score plot of Group A (HC, pink circles) and Group B (pSCH, blue circles) cows. Each dot represents an individual dairy cow sample. The substantial overlap of samples in the PCA plot indicates that the proteomic method employed is robust, highly reproducible, and stable. Panel (**B**) presents the OPLS−DA score plot, with pink representing Group A and blue representing Group B; ellipses denote the 95% confidence intervals. The complete separation of the two groups indicates a significant difference in protein expression profiles between them. Panel (**C**) displays the permutation test results: the original model had R^2^Y = 0.878 and Q^2^ = 0.313; all permuted Q^2^ values were lower than the original Q^2^, confirming the absence of overfitting.

To systematically evaluate the overall differences in protein expression profiles between the two groups, multivariate statistical analyses were performed using principal component analysis (PCA) and orthogonal partial least squares discriminant analysis (OPLS-DA), and the reliability of the OPLS-DA model was validated by permutation testing. The PCA score plot (Figure 1A) showed a certain tendency toward separation between the proteomic profiles of subclinically hypocalcemic cows and healthy cows, with the first two principal components collectively explaining 51.09% of the protein expression variance and some overlap between the two groups. The OPLS-DA score plot (Figure 1B) demonstrated that the protein expression profiles of the pSCH and healthy groups were clearly distinguished, with each group forming a distinct cluster and marked between-group differences in protein expression characteristics. The permutation test (Figure 1C) yielded an R^2^Y of 0.878 and a Q^2^ of 0.313 for the OPLS-DA model. The Q^2^ value, although moderate in magnitude, is distinctly greater than zero, which is the critical threshold for meaningful predictive ability. This indicates that the model possesses sufficient discriminative power to reliably differentiate between Group A and Group B.

### 3.2. Analysis of Differential Protein Expression

**Figure 2:**
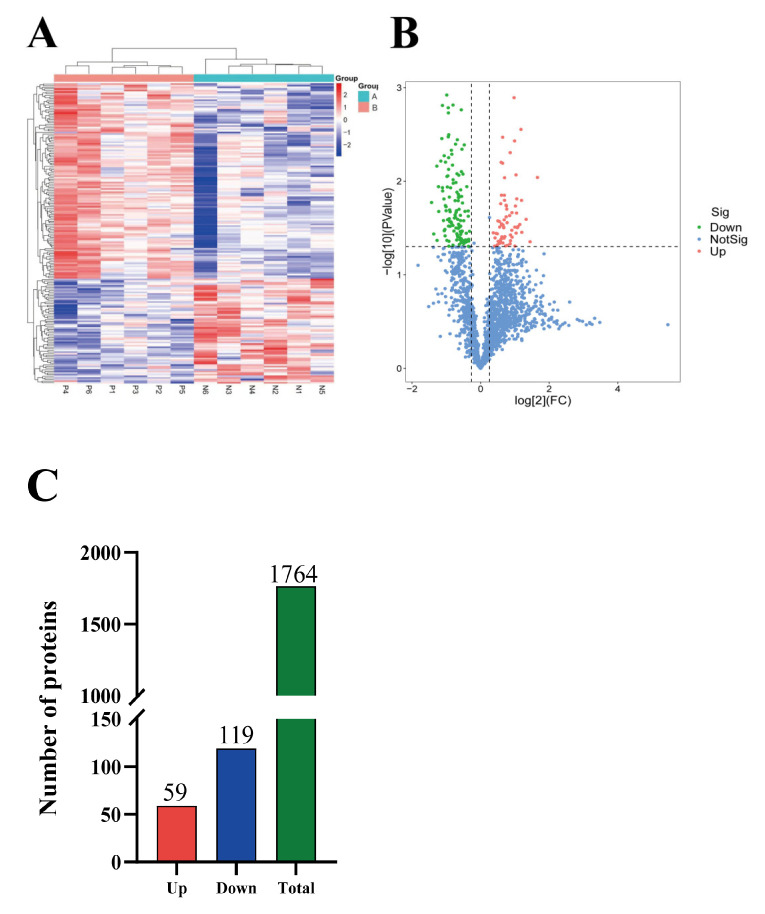
Cluster heatmap, volcano plot, and bar chart of differentially expressed protein counts for the pSCH and healthy control (HC) groups. In panel (**A**), the *x*-axis represents the samples (Group A: N1–N6; Group B: P1–P6) and the *y*-axis represents the differentially expressed proteins. The color gradient indicates normalized protein expression values, with red denoting high expression and blue denoting low expression. Both samples and proteins were subjected to hierarchical clustering, revealing distinctly different expression patterns between the two groups. In panel (**B**), the *x*-axis represents the log_2_-transformed fold change (log_2_ FC) in protein expression, and the *y*-axis represents the −log_10_-transformed *p* value.Vertical dashed lines represent the fold-change threshold (|log_2_FC| > log_2_1.2), and the horizontal dashed line indicates the significance threshold (*p* < 0.05). Proteins falling outside these thresholds were considered significantly differentially expressed. A total of 59 significantly up-regulated proteins (red), 119 significantly down-regulated proteins (green), and 1764 proteins with no statistically significant difference (blue) are shown. In panel (**C**) showing the numbers of up-regulated, down-regulated, and total differentially expressed proteins. A total of 1764 proteins were identified in this study, of which 178 were significantly differentially expressed, comprising 59 up-regulated and 119 down-regulated proteins.

To identify differentially expressed proteins between Group A and Group B, a *t*-test was applied to assess the significance of between-group differences, and differentially expressed proteins were filtered according to the aforementioned thresholds. The overall distribution of these proteins was displayed using a volcano plot, and hierarchical clustering was performed on the differentially expressed proteins. The cluster heatmap (Figure 2A) showed that the protein expression profiles of pSCH cows and healthy cows were clearly distinguishable: samples from each group formed a distinct cluster, with consistent intra-group protein expression patterns, and the differentially expressed proteins between groups exhibited marked clustering characteristics. The volcano plot (Figure 2B) revealed that, compared with healthy cows, a total of 178 significantly differentially expressed proteins were identified in pSCH cows, among which 59 were significantly up-regulated and 119 were significantly down-regulated, while the expression of the other 1586 proteins showed no statistically significant differences. The key differential proteins between the two groups of cows are listed in Table 2.

### 3.3. Differential Protein Function Enrichment Analysis

**Figure 3:**
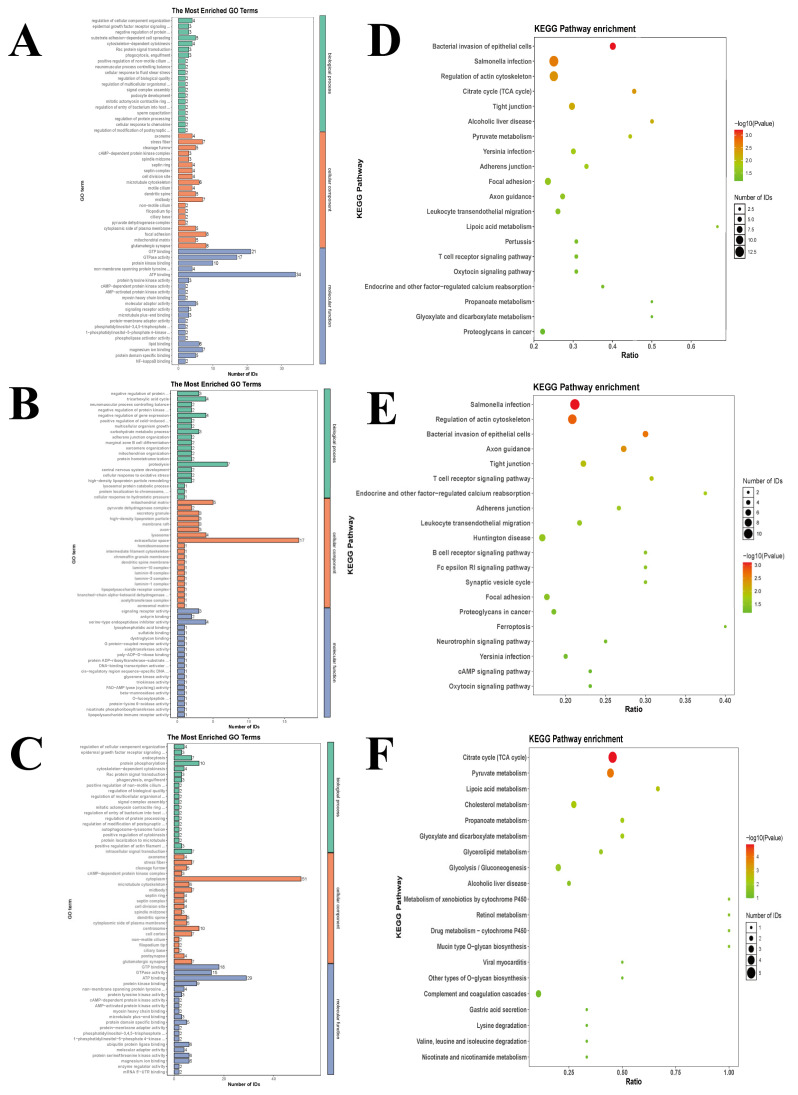
GO functional enrichment and KEGG pathway analysis of differentially expressed proteins between pSCH dairy cows and healthy dairy cows. GO enrichment analysis of all differentially expressed proteins in serum samples from the two experimental groups (**A**). GO enrichment results for down-regulated differentially expressed proteins (**B**). GO enrichment results for up-regulated differentially expressed proteins (**C**). KEGG pathway enrichment results for all differentially expressed proteins (**D**). KEGG pathway enrichment results for down-regulated differentially expressed proteins (**E**). KEGG pathway enrichment results for up-regulated differentially expressed proteins (**F**).

Based on the bioinformatics analysis of the 178 differentially expressed proteins (including GO, KEGG, and pathway analyses), together with a review of the relevant literature and data, a total of 22 differentially expressed proteins were identified as potentially associated with the pathogenesis of pSCH. The GO functional enrichment and KEGG pathway analyses of the differentially expressed proteins are presented in Figure 3.

The significantly enriched pathways among the differentially expressed proteins in serum samples were endocrine and other factor-regulated calcium reabsorption, regulation of actin cytoskeleton, the TCA cycle, and lipoic acid metabolism. 

Using the KEGG pathway database, we searched for significantly enriched pathways among the differentially expressed proteins. Three proteins—AP2A2, AP2B1, and AP2A1 (all down-regulated)—were involved in endocrine and other factor-regulated calcium reabsorption. Twelve proteins—MYL12B, MYL9, RHOA, KRAS, CYFIP1, CDC42, RDX, PIP4K2A, VAV1, and PIP4K2B (down-regulated), along with ACTB and ARPC5L (up-regulated)—were involved in the regulation of actin cytoskeleton pathway. Five proteins—FH, SUCLG2, CS, DLD, and DLAT (all up-regulated)—were involved in the TCA cycle pathway, among which DLD and DLAT (both up-regulated) also participated in the lipoic acid metabolism pathway. 

To elucidate the biological functions and signaling pathways involving the differentially expressed proteins, Gene Ontology (GO) functional enrichment analysis and Kyoto Encyclopedia of Genes and Genomes (KEGG) pathway enrichment analysis were performed on all differentially expressed proteins, the up-regulated proteins, and the down-regulated proteins, respectively. 

The GO functional enrichment results showed that the differentially expressed proteins encompassed multiple functional categories, including immune regulation, energy metabolism, and cytoskeletal integrity assembly, with significantly enriched terms identified at the levels of biological process, cellular component, and molecular function. The KEGG pathway enrichment results revealed that the differentially expressed proteins were significantly enriched in physiological regulatory pathways such as the tricarboxylic acid (TCA) cycle and calcium reabsorption pathway. 

**Figure 4:**
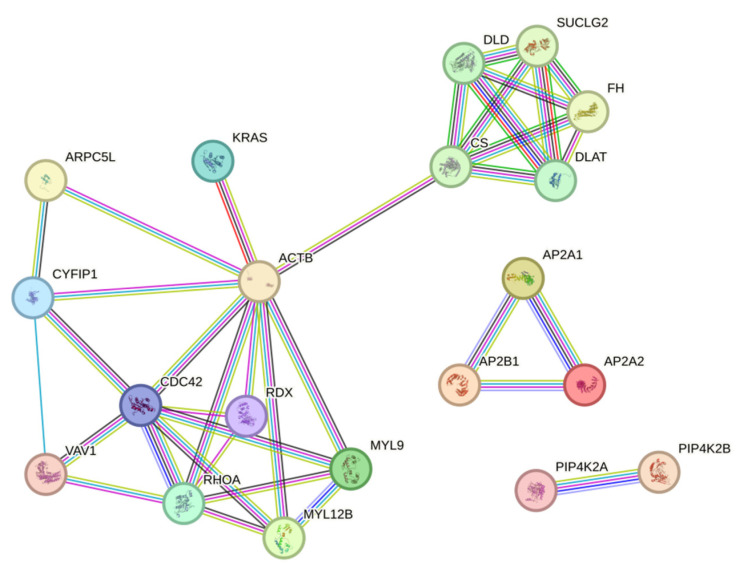
The network interaction diagram of differential expression proteins. In this network, each node represents a protein. Node colors indicate the direction of expression change: red nodes denote up-regulated proteins and blue nodes denote down-regulated proteins in the pSCH group relative to the HC group (fold change > 1.2, *p* < 0.05). The fill pattern of nodes indicates structural information: solid-filled nodes have known or predictable three-dimensional structures, whereas hollow nodes do not. Edges (lines) represent interactions between proteins. Different edge colors correspond to distinct types of evidence supporting the interaction, including curated databases, experimentally determined interactions, gene co-expression, gene neighborhood, gene fusion, text mining, and protein homology, as annotated by the STRING database. Edge thickness is proportional to the confidence score of the interaction, with thicker lines indicating higher confidence (e.g., higher combined score).

Protein–protein interaction (PPI) network analysis of the differentially expressed proteins revealed that the network formed four functional modules. The module centered on CDC42 contained proteins such as ACTB, RHOA, and MYL9 and was primarily involved in cytoskeleton regulation. Additional modules were associated with clathrin-mediated endocytosis, phosphatidylinositol metabolism, and the tricarboxylic acid (TCA) cycle, respectively. The results are shown in Figure 4. 

## 4. Discussion

The endocrine and other factor-regulated calcium reabsorption pathway was the most significantly altered calcium metabolism-related pathway in this study, in which three core subunits of adaptor protein complex 2(AP-2)—AP2A1, AP2A2, and AP2B1—were all significantly down-regulated. The AP-2 complex is a key regulatory molecule in clathrin-mediated endocytosis: AP2A1 and AP2A2, as α-adaptin isoforms, are responsible for recognizing the conserved YXXΦ tyrosine-based endocytic motif in the cytoplasmic tails of membrane proteins [13]; AP2B1, the β subunit, binds the clathrin-heavy chain to initiate vesicle budding and membrane internalization. Together, these three subunits govern the abundance of functional proteins on the cell membrane [14]. In vitro studies have demonstrated that the AP-2 complex recognizes the endocytic motif of the renal apical membrane calcium channel TRPV5, mediating its clathrin-dependent endocytosis and recycling, and is a critical factor in maintaining the stability of TRPV5 membrane localization and ensuring renal calcium reabsorption [15]. The synchronous down-regulation of all three subunits observed in this study would directly lead to disrupted TRPV5 membrane localization, impaired recycling, and aberrant degradation, thereby weakening the efficiency of renal calcium reabsorption from the glomerular filtrate. This may result in persistently increased urinary calcium excretion and impair the rebound of blood calcium through postpartum homeostatic regulation, which constitutes the core molecular basis for the persistently low blood calcium in pSCH cows, and is highly consistent with previous findings that defective renal calcium reabsorption mediates hypocalcemia [16]. 

The regulation of actin cytoskeleton pathway showed an overall down-regulation trend. The actin cytoskeleton is the core structural network of eukaryotic cells, and in addition to maintaining cell morphology, it is deeply involved in physiological processes such as ion channel membrane anchoring, calcium signaling, smooth muscle contraction, and transmembrane transport [17]. Among the differentially expressed proteins, MYL12B and MYL9 encode non-muscle myosin II regulatory light chain and smooth muscle myosin regulatory light chain, respectively, which are core effector molecules coupling calcium signals to the actin–myosin contractile machinery [18,19]: MYL12B activates myosin ATPase activity through phosphorylation, driving actin filament sliding and participating in vascular smooth muscle contraction and the assembly of calcium signaling complexes [20]; MYL9 governs smooth muscle cell contraction and maintains tissue perfusion, while mammary blood flow directly determines the efficiency of calcium transport into milk [21,22]. Although MYL12B (non-muscle myosin II regulatory light chain) and MYL9 (smooth muscle myosin regulatory light chain) both belong to the myosin II regulatory light chain family, they differ fundamentally in their activation mechanisms and cellular functions: MYL12B predominantly responds to signaling by Rho-associated kinase (ROCK) and myosin light chain kinase (MLCK) in non-muscle cells, where reversible phosphorylation at Ser19/Thr18 regulates the hexameric assembly and contractile activity of non-muscle myosin II, thereby driving dynamic cytoskeletal remodeling processes including cell migration, stress fiber formation, and cytokinesis; in contrast, MYL9 relies specifically on Ca^2+^/calmodulin-activated MLCK in smooth muscle cells and establishes an antagonistic balance between Ser19 phosphorylation and myosin phosphatase activity to generate sustained tonic contraction, maintaining the physiological tone of blood vessels, airways, and visceral organs. Thus, the core distinction lies in the fact that MYL12B executes rapid and reversible cytoskeletal remodeling to support cell motility and morphological changes, whereas MYL9 mediates homeostatic smooth muscle contraction to regulate organ tone [23,24,25]. This pathway exhibits a bidirectional causal relationship with pSCH: on the one hand, calcium ions are essential activators for myosin light chain phosphorylation, and sustained low blood calcium directly inhibits myosin activation and cytoskeletal contractile function, making the down-regulation of pathway proteins a direct downstream effect of hypocalcemia; on the other hand, weakened vascular smooth muscle contraction leads to insufficient mammary tissue perfusion, impeding the delivery of calcium to the mammary gland. Superimposed on the continuously increasing calcium consumption during peak postpartum lactation, the supply–demand gap progressively widens, further aggravating the hypocalcemic state. This creates a vicious cycle of “hypocalcemia→contractile dysfunction→inadequate calcium supply” that jointly drives the progression of pSCH. 

In this study, the TCA cycle and lipoic acid metabolism pathways were significantly up-regulated and enriched, with core proteins such as citrate synthase (CS), DLAT, and DLD all highly expressed. The TCA cycle is the core pathway for cellular ATP production [26,27], in which CS is the first irreversible rate-limiting enzyme that determines the overall flux of the cycle [28]. DLAT and DLD together constitute the core components of the pyruvate dehydrogenase complex and also participate in the redox cycling of the lipoic acid prosthetic group, directly affecting mitochondrial energy metabolism efficiency [29,30]. SUCLG2 and FH are involved in substrate-level phosphorylation and oxaloacetate regeneration, respectively, maintaining the operation of the cycle [31,32]. The up-regulation of these pathways does not reflect mitochondrial hyperfunction but is essentially a compensatory response to impaired energy metabolism under hypocalcemia. Previous studies have confirmed that key rate-limiting enzymes of the TCA cycle are calcium-dependent, and a decrease in mitochondrial matrix calcium concentration directly inhibits their catalytic activity. Under sustained hypocalcemia, the organism up-regulates the expression of core enzyme proteins and enhances lipoic acid metabolism efficiency to compensate for the insufficient ATP production resulting from decreased enzyme activity [33,34]. When milk production demand is relatively low in the early postpartum period, this compensatory mechanism can still maintain basal energy supply, which is consistent at the protein expression level with the AMPK-mediated low-calcium compensation mechanism proposed by Hardie [35]. However, as milk yield surges postpartum, energy demand increases explosively, the compensatory limit is exceeded, and ATP production becomes severely insufficient, leading to functional decline in active energy-consuming processes such as renal calcium reabsorption and mammary calcium transport, ultimately driving the occurrence and progression of pSCH. 

This study has several limitations: the sample size was small (n = 6 per group), and although rigorous multivariate statistical corrections were applied, large-scale cohort validation is still required; serum proteomics cannot directly reflect specific changes in target organs such as the kidney and mammary gland; and the cross-sectional association design cannot establish causality. Future studies may include proteomic analyses of kidney and mammary gland tissues, cellular-level functional validation of key proteins, and screening for early diagnostic markers of hypocalcemia. A limitation of this study is that the differentially expressed proteins were not experimentally validated, and this remains to be addressed in future research. In addition, the protein targets identified in this study offer both theoretical and practical value for periparturient calcium nutrition regulation and the prevention of pSCH. Among the 178 differentially expressed proteins identified in this study, the down-regulated AP-2 subunits (AP2A1, AP2A2, AP2B1) and cytoskeletal regulators (MYL12B, MYL9, RHOA, CDC42) are the most promising candidates as early biomarkers for pSCH, as they directly implicate impaired renal calcium reabsorption and compromised vascular contractile function, respectively. Their diagnostic value lies in potentially distinguishing pSCH from transient subclinical hypocalcemia before secondary metabolic complications arise, while their predictive utility may guide timely calcium supplementation and mitigate disease progression. However, clinical translation requires independent large-scale cohort validation, establishment of sensitivity- and specificity-based cut-off values, and development of cost-effective on-farm detection platforms. Currently, the lack of validation across different herds and farm conditions, together with the absence of standardized high-throughput assays, constitutes a major barrier to routine implementation, and future prospective studies are needed to confirm whether biomarker-guided intervention improves long-term outcomes. 

## 5. Conclusions

In this study, 4D-DIA quantitative proteomics was employed to analyze the differential expression profiles of serum samples from six healthy Holstein cows and six pSCH cows, and a total of 178 significantly differentially expressed proteins were identified, including 59 up-regulated and 119 down-regulated proteins. Bioinformatics enrichment analysis revealed that the differentially expressed proteins were predominantly enriched in four core pathways: the down-regulated pathways of endocrine and other factor-regulated calcium reabsorption and regulation of actin cytoskeleton, which mediate impaired renal calcium reabsorption and disrupted cellular calcium–contraction coupling, respectively; and the up-regulated pathways of the TCA cycle and lipoic acid metabolism, which preliminarily characterized the serum protein profiles and identified potential pathways and biomarkers. In conclusion, pSCH is a systemic metabolic disorder arising from the dysregulation of multiple physiological pathways. 

## Figures and Tables

**Table 1:** Information of pSCH and HC cows.

Background Information	pSCH (n = 6)	HC (n = 6)	*p* Value
Age	2.96 ± 0.10	2.88 ± 0.13	0.636
Parity	1.60 ± 0.09	1.52 ± 0.29	0.797
BCS	2.82 ± 0.05	2.86 ± 0.03	0.508
Milk yield (kg/d)	26.83 ± 0.22	26.78 ± 0.23	0.878
DIM (d)	6.62 ± 0.27	6.58 ± 0.24	0.914
1d (mmol/L)	1.72 ± 0.06	1.95 ± 0.07 *	0.032
2d (mmol/L)	1.97 ± 0.05	2.25 ± 0.06 **	0.0049
4d (mmol/L)	2.12 ± 0.07	2.35 ± 0.05 *	0.023

Note: * *p* < 0.05; ** *p* < 0.01. Values are presented as mean ± standard deviation (SD). Ranges (Min–Max) for baseline parameters: pSCH—Age: 2.86–3.06. Parity: 1–2. BCS: 2.5–3.0. Milk yield: 26.61–27.05. DIM: 6.35–6.89. HC—Age: 2.75–3.01. Parity: 1–2. BCS: 2.5–3.0. Milk yield: 26.55–27.01. DIM: 6.34–6.82.

**Table 2:** Key proteins associated with pSCH.

No.	ID	Proteins	Protein Full Name	Trend	Pathway
1	Q0VCK5	AP2A2	AP-2 complex subunit alpha-2	↓	Endocrine and other factor-regulated calcium reabsorption
2	Q08DS7	AP2B1	AP-2 complex subunit beta	↓	
3	E1BMW2	AP2A1	AP-2 complex subunit alpha	↓	
4	Q5E9E2	MYL12B	Myosin regulatory light polypeptide 9	↓	Regulation of actin cytoskeleton
5	Q1LZF9	MYL9	Myosin, light chain 9, regulatory	↓	
6	P61585	RHOA	Transforming protein RhoA	↓	
7	Q1LMP2	KRAS	multidrug efflux system outer membrane porin	↓	
8	E1BN47	CYFIP1	Cytoplasmic FMR1-interacting protein	↓	
9	Q2KJ93	CDC42	Cell division control protein 42 homolog	↓	
10	Q32LP2	RDX	Radixin	↓	
11	F1MHR4	PIP4K2A	Phosphatidylinositol 5-phosphate 4-kinase type-2 alpha	↓	
12	Q08DN7	VAV1	Proto-oncogene vav	↓	
13	F1MHT2	PIP4K2B	phosphatidylinositol-5-phosphate 4-kinase, type II, beta	↓	
14	P60712	ACTB	Actin, cytoplasmic 1	↑	
15	Q5E963	ARPC5L	Actin-related protein 2/3 complex subunit 5-like protein	↑	
16	Q148D3	FH	Fumarate hydratase, mitochondrial	↑	TCA cycle
17	Q3MHX5	SUCLG2	Succinate–CoA ligase [GDP-forming] subunit beta, mitochondrial	↑	
18	Q29RK1	CS	Citrate synthase, mitochondrial	↑	
19	F1N206	DLD	Dihydrolipoyl dehydrogenase, mitochondrial	↑	
20	P11180	DLAT	Dihydrolipoyllysine-residue acetyltransferase component of pyruvate dehydrogenase complex	↑	
21	F1N206	DLD	Dihydrolipoyl dehydrogenase, mitochondrial	↑	Lipoic acid metabolism
22	P11180	DLAT	Dihydrolipoyllysine-residue acetyltransferase component of pyruvate dehydrogenase complex	↑	

Note: “↓” Compared to the HC group, the pSCH group is decreased. “↑” Compared to the HC group, the pSCH group is increased.

## Data Availability

The data presented in this study are available on request from the corresponding authors.

## Supplementary Materials

The following supporting information can be downloaded at: https://www.mdpi.com/article/10.3390/ani16152378/s1, Table S1. (Abbreviations are listed in alphabetical order with their corresponding full names.)

## Abbreviations

Abbreviations are listed in alphabetical order with their corresponding full names.

4D-DIA	4-Dimensional Data-Independent Acquisition
AMPK	AMP-Activated Protein Kinase
AP-2	Adaptor Protein Complex 2
BCS	Body Condition Score
DIM	Days (or Day) in Milk
dSCH	Delayed Subclinical Hypocalcemia
HC	Healthy Control
HPLC	High-Performance Liquid Chromatography
KEGG	Kyoto Encyclopedia of Genes and Genomes
KNN	K-Nearest Neighbor
LC-MS/MS	Liquid Chromatography–Tandem Mass Spectrometry
MLCK	Myosin Light Chain Kinase
OPLS-DA	Orthogonal Partial Least Squares Discriminant Analysis
PCA	Principal Component Analysis
PPI	Protein–Protein Interaction
pSCH	Persistent Subclinical Hypocalcemia
ROCK	Rho-Associated Kinase
TCA	Tricarboxylic Acid (cycle)
TMR	Total Mixed Ration
TRPV5	Transient Receptor Potential Vanilloid 5
tSCH	Transient Subclinical Hypocalcemia
