# Supplementary material for: Proteomic Analysis of Dairy Cows with Persistent Subclinical Hypocalcemia
Source: Animals (Basel). 2026 Aug 3;16(15):2378. doi: 10.3390/ani16152378 (PMC13465629; doi:10.3390/ani16152378)
Supplement: Supplementary file 1 [file animals-16-02378-s001.zip › animals-4445718-supplementary.pdf]

Table S1.

Cow ID	Parity	Day 1 (mmol/L)	Day 2 (mmol/L)	Day 4 (mmol/L)	Classification	Final Selection
P1	1	1.64	1.91	2.03	pSCH	Selected
P2	2	1.67	1.92	2.06	pSCH	Selected
P3	1	1.71	1.96	2.10	pSCH	Selected
P4	2	1.73	1.98	2.14	pSCH	Selected
P5	2	1.76	2.02	2.18	pSCH	Selected
P6	2	1.77	2.03	2.20	pSCH	Selected
N1	1	2.15	2.17	2.28	HC	Selected
N2	1	2.16	2.20	2.31	HC	Selected
N3	1	2.18	2.23	2.33	HC	Selected
N4	2	1.87	2.27	2.37	HC	Selected
N5	2	1.95	2.30	2.39	HC	Selected
N6	2	2.04	2.33	2.42	HC	Selected
R1	2	1.70	2.10	2.25		Excluded
R2	2	1.65	2.08	2.30		Excluded
R3	1	1.72	2.16	2.28		Excluded
R4	1	1.68	2.17	2.35		Excluded
R5	2	1.75	2.05	2.40		Excluded
R6	1	1.60	2.18	2.33		Excluded
R7	2	1.74	2.00	2.26		Excluded
R8	2	1.63	2.22	2.38		Excluded
R9	2	1.77	2.16	2.42		Excluded
R10	1	1.80	2.20	2.10		Excluded
R11	2	1.85	2.25	2.05		Excluded
R12	2	1.90	2.30	2.15		Excluded
R13	1	1.82	2.18	2.08		Excluded
R14	1	1.88	2.28	2.12		Excluded
R15	2	1.92	2.32	2.02		Excluded
R16	1	1.78	2.22	2.18		Excluded
R17	1	1.83	2.26	2.06		Excluded
R18	1	1.86	2.24	2.04		Excluded
